# Assessment of Virtual Peer Learning by Peer Feedback: A Pilot Project

**DOI:** 10.7759/cureus.30596

**Published:** 2022-10-23

**Authors:** Nachappa Sivanesan Uthraraj, Nina Mary Charles, Sean M Garcia, Annis Maatough, Fitzgerald Anazor, Sumathi Krishnamurthy, Laya Manasa Sriraam, Kannaki Chettiakkapalayam Venkatachalam, Jai Relwani

**Affiliations:** 1 Trauma and Orthopedics, William Harvey Hospital, Ashford, GBR; 2 Medical and Surgical Research, Thamarai Health Care, Coimbatore, IND; 3 Critical Care, Nottingham University Hospital, Nottingham, GBR; 4 General Surgery, University College of Medical Sciences (UCMS) and Guru Teg Bahadur (GTB) Hospital, New Delhi, IND; 5 Otolaryngology, Kanchi Kamakoti Childs Trust Hospital, Chennai, IND; 6 Reproductive Medicine, The Fertility Center, Kovai Medical Center and Hospital (KMCH), Coimbatore, IND; 7 Reproductive Genetics, Sivameds Fetal Medicine and Fertility Research (SIMFFER) Foundation, Coimbatore, IND

**Keywords:** likert scale, small group teaching, virtual learning, peer feedback, peer-assisted learning

## Abstract

Introduction

Virtual learning has become the preferred modality for health education during and after the coronavirus disease 2019 (COVID-19) pandemic. Peer learning is gaining a lot of significance lately and has been successfully tested in various settings. We combined virtual delivery of health education with peer-assisted learning and evaluated the effectiveness using peer-generated feedback and tested the effectiveness of the model for different cohorts.

Methods

We performed this study as part of a formal educational course on Health Professions Education. The educators were volunteers from different informal multi-disciplinary groups, working in varied healthcare settings, globally. This involved eight teaching sessions which were delivered virtually and the feedback was recorded as responses to six items (questions), which the learners graded on the Likert scale. The average for each item and the larger domains was then calculated and analyzed.

Results

The feedback was provided by all the participants (53/53). In the feedback received item-wise, the best average rating was for legibility of the slides (4.8). The least rating was for adequate checking and assessment of prior knowledge (4.2). In terms of the broader domains, the best feedback was for the teaching material (4.6) and the lowest was for the planning of the sessions (4.4). Overall, the ratings for the domains and the items were above 3 on a scale of 1-5.

Conclusions

Virtual delivery of healthcare education, facilitated by peer-assisted learning, is an effective model for health education when delivered for a small group, as evidenced by the overall peer feedback. This model can be tested for larger cohorts in the future.

## Introduction

Virtual learning is a modality that has increased in significance and application over the last few years. Virtual or online learning as described by Howlett et al. is the use of electronic technology and media to facilitate and improve learning and teaching, involving communication between the learner and the educator, with respect to the online content [1]. It is shown to have similar outcomes to conventional face-to-face teaching [2]. The need for virtual teaching was highlighted internationally because of the constraints due to the coronavirus disease 2019 (COVID-19) pandemic, which are still in place, in some parts of the world. Peer observation of teaching and subsequent feedback provides qualitative evidence of its effectiveness [3]. The incorporation of the same at a macro level can contribute to the professional development of educators [4]. Peers in clinical teaching are individuals from similar social groupings, not essentially professionally trained as teachers, but who help each other to learn and learn themselves in the process [5]. Peer-assisted learning or peer learning is when the teaching session is delivered by a peer to a peer or peers [6]. Peer feedback is when peers who have observed or participated in the teaching activity provide feedback to the educator who is a peer as well [2]. Peer-assisted learning is a style, which improves the learning environment and helps the participants in their professional identity formation. It is more effective when introduced in the curriculum for easy topics [7,8]. The objective of this study was to evaluate the effectiveness of this model, which combines peer learning in a virtual environment, with the score from the peer-generated feedback of the educators by the learners. This project is intended to improve the general quality of healthcare education. This pilot model, incorporating the three elements (peer learning, peer feedback, and the virtual environment), was used in small group teaching, internationally, and for multidisciplinary healthcare cohorts. The effectiveness of this model across the different sessions was then analyzed.

## Materials and methods

The teaching was delivered by six peer educators. There were eight sessions facilitated over eight months. The sessions/modules taught were as follows: metastatic disease of the spinal cord, assessment and management of pyrexia, heart-rate variations, venous access, stoma care, post-operative monitoring in acute surgery, nutritional support for the surgical patient, and internal derangements of the knee joint. The teaching material for the session was developed by peer educators. The learners and the peer educators were healthcare professionals from multidisciplinary backgrounds. The modules as enlisted above were not restricted to a particular specialty and were chosen at a basic level, relevant to patient-based care in any district general hospital. The learners were working in different capacities, in different international healthcare systems. Invitations were sent to potential learners as identified by the peer educators and willing participants were registered for this project. Potential learners were healthcare professionals who had access to virtual learning and had scope for improvement from the learning. The platform for virtual teaching (Zoom Video; San Jose, CA: Zoom Video Communications Inc.) was identified, after checking the convenience and accessibility for everyone. The knowledge of the learners was assessed informally before each session and found to be variable. The length of each teaching session was set at 40 min in accordance with the average face-to-face lecture sessions in medical schools. The Microsoft PowerPoint application (Redmond, WA: Microsoft Corporation) was used to prepare and deliver the teaching material as slides. The teaching slides consisted of text and pictures. There were assessments in the form of mini quizzes during the session to assess comprehension and to encourage learner engagement during the session. The peer feedback was collected in a written format, by way of a structured feedback form (Appendices), sent to the participants by email and all the participants responded within one week. There was no specific feedback model used in this study. The effectiveness of the teaching was evaluated against clearly defined learning objectives and outcomes, unique for each session, using a Likert scale rating (1-5), with 1 being the least and 5 being the best. As this was being done as a pilot model, we were not mandated to validate the feedback questionnaire. The items assessed in the feedback were as follows: achievement of the learning outcome, adequacy of checking and assessment of prior knowledge, engagement of the facilitator and introduction to the session (opening of the session by the facilitator and "breaking-ice"), adequacy of modalities used in the teaching session, legibility and comprehension (in terms of the ease of following) of the teaching slides, breaking down of concepts and building up from previous knowledge. This was then grouped into the following domains: planning of the session, communication skills, and the quality of the teaching material (Appendices). There was also a free text box for any other qualitative comments. The grading on the Likert scale was computed for the average, item-wise and domain-wise. Verbal consent was obtained from all the peer learners and educators who participated in this project. This project was registered with an institutional ethics committee.

## Results

There were a total of 53 participants from multidisciplinary backgrounds, who had accepted the invitation and participated. Feedback was obtained through a structured qualitative feedback form. On the Likert scale, the average scores for the different items (questions) were as follows: achievement of learning outcomes - 4.6, adequate checking and assessment of prior knowledge - 4.2, opening of the session by the facilitator and "breaking-ice" - 4.5, adequacy of the modalities used in the teaching session - 4.3, legibility and ease in terms of following the slides - 4.8, and breaking down of concepts and building up from previous knowledge - 4.6 (Table 1). The average Likert score for the three domains was as follows: planning of the session - 4.4, communication skills - 4.5, and the quality of the teaching material - 4.5 (Table 2). The highest average rating was for legibility and comprehension (ease with which the slides could be followed) of the slides - 4.8 and the lowest was for checking and assessment of previous knowledge - 4.2. In terms of the domains, the highest average rating was for the quality of the teaching material - 4.6 and the lowest for the planning of the session - 4.4. There was feedback in the form of free text, from two of the peers - the need for more simulated case scenarios and the limited scope for the use of additional modalities was emphasized. The images used in the PowerPoint slides were appreciated.

**Table 1:** Question-wise means for each teaching session.

	Achievement of learning outcomes	Adequacy of checking and assessment of prior knowledge	Opening of the session by the facilitator and "breaking-ice"	Adequacy of modalities used in the teaching session	Legibility and ease in terms of following the slides	Breaking down of concepts and building up from previous knowledge
Metastatic disease of the spinal cord	5	4.4	5	4.8	4.8	4.8
Assessment and management of pyrexia	5	4	5	4.4	5	4.4
Heart rate variations	5	4.3	4.8	4.5	4.8	4.8
Venous access	4.8	4.4	4.8	4.2	5	4.4
Stoma care	4.5	3.5	4.1	4.4	5	4.6
Post-operative monitoring in acute surgery	3.8	4.4	3.6	4.1	4.6	4.6
Nutritional support in gastrointestinal surgery	4.5	4.6	4.5	4.3	4.9	4.6
Internal derangement of the knee joint	4.3	3.7	4.3	4.1	4.6	4.6
Mean	4.6	4.2	4.5	4.3	4.8	4.6

**Table 2:** Domain-wise means for each teaching session.

	Planning of the session	Communication skills	Quality of the teaching material
Metastatic disease of the spinal cord	4.7	5	4.8
Assessment and management of pyrexia	4.5	5	4.6
Heart rate variations	4.7	4.8	4.7
Venous access	4.6	4.8	4.5
Stoma care	4	4.1	4.7
Post-operative monitoring in acute surgery	4.1	3.6	4.4
Nutritional support in gastrointestinal surgery	4.6	4.5	4.6
Internal derangement of the knee joint	4	4.3	4.4
Mean	4.4	4.5	4.6

## Discussion

This project could demonstrate qualitatively and by way of peer feedback through the Likert ratings that small group peer-assisted learning, on a virtual platform, can be a successful and comparable model of healthcare education delivery, to conventional methods.

Feedback helps make the learning experience better for both the learner and the educator. In our study, this is evident from the responses to the three domains which comprehensively assess the learning experience. Peer feedback can inform the educator about the professionalism of the session and contribute to their Professional Development Plan (PDP). There can be barriers for learners, especially formal feedback from students, due to social discomfort, sense of responsibility, and task difficulty [9,10]. The necessity to provide feedback of the learning environment as a whole encourages learners to be more engaged and active with the learning process [11]. Feedback literacy, as described by Tripodi et al., places the onus on the receiver to act on the feedback and a framework has been developed for further development, on giving and receiving feedback [12]. In our pilot project, we found that there was robust engagement from the learners, as judged during the session and from the structured feedback received from all the participants. This helped the educator prepare for future sessions and to include the salient points in their PDP. As the groups were informal and formed amongst peers, there was minimal to no hesitancy in providing honest and accurate feedback.

Peer learning can result in a more engaged and collaborative learning environment [13]. In our study, the educators were able to match the course content better to the needs of the learner as demonstrated by the feedback. This resulted in a collaborative learning environment. Peer-assisted learning can be a suitable mode for easy topics [8]. Umbreen et al. conducted a study, where anatomy was taught by the peer-learning model, over a 32-week period and found that the communication, teaching skills, and confidence of the educators increased considerably [14]. This methodology, when employed to foundation doctors in the United Kingdom, was found to better match the educational content with the learning needs and experience of learners and facilitated active learning - a learning environment in which questions and conjectures were safely shared [15,16]. Varghese et al. were able to demonstrate, through qualitative data, for small groups, in the dental surgery undergraduate curriculum, that peer-assisted learning increased confidence, developed skills for the learners, and build confidence in the educators when compared with conventional learning [17]. In the specific context of surgical skill-based training, peer-assisted learning provided optimal learning opportunities within the established curriculum, as compared to the conventional hierarchical teaching model [18]. In the pilot study by Elshami et al., using peer learning, radiography learners felt that their learning experience was enriched, helping them better prepare for their exams [19]. Near-peer learning is an adjunct to peer learning, where peers support, but do not necessarily direct the session. When near-peer learning sessions were compared to e-learning for the same course content, the near-peer learning cohort demonstrated better performance statistically [20]. In our project, we felt that the learners would be better prepared and more confident for patient encounters or simulation-based expansion of the course content, after peer-assisted learning, as compared to conventional models. The educators observed that their confidence and teaching skills improved, though this was not assessed formally. We did not use near-peer learning, but that can be included in future sessions for technically demanding course material that needs expert guidance.

The COVID-19 clinical rounds initiative undertaken in the United States, in response to the COVID-19 pandemic, was a huge success demonstrating the feasibility of delivering virtual teaching to a large group, as a rapid response [21]. Domb et al. successfully developed a model based on the Zoom platform, for clinical teaching and observation, involving the supervisor, learner, and real or simulated patients. This model enabled the supervisor to observe patient-learner encounters and provide constructive feedback. Different platforms for delivering virtual teaching were compared and the Zoom platform was found to have higher acceptability, recognition, and ease of use [22]. Objective structured clinical examinations and grand rounds were trialed with the Zoom platform and found to be successful as learning environments in these settings [8,15]. In this project, of all the platforms available, the educator and the learners of this project felt that the Zoom conferencing platform was the appropriate choice due to familiarity, ease of use, and supportive features. Time restriction, which is a built-in feature in the Zoom application, was inconsequential, as all the sessions were timed within the permitted window. We could appreciate the time saved in preparation and logistics, by virtual teaching, which could be applied to teaching sessions planned at short notice. The comfort in the learning environment was also appreciable as evidenced in the feedback responses.

The strengths of the study are the testing of this model internationally and in multidisciplinary settings in healthcare by various healthcare peer educators that validates its universality and ease of use. The limitations of this study are that the feedback was received using only one structured scale, it was not tested for the undergraduate curriculum, and not applied to large-group teaching. The future direction would be to mitigate these and to obtain feedback using different scales and modes for larger groups.

## Conclusions

This project combines peer-assisted learning, peer feedback, and a virtual learning environment in the same session for each of the eight sessions. This study demonstrates the overall acceptance of this model. The presentation and organization of the learning material received the highest ratings, while planning of the sessions scored the lowest. We were able to identify the particular areas that were universally deficient in all the sessions and the strengths as well. The feedback obtained will help both the learners and the educator in planning future sessions, including for large groups. This project helped the educators reflect and devise plans to deliver similar sessions, for different learner cohorts and other themes as well. The multidisciplinary nature of the audience also helped to obtain a 360° view of this model. This study will hopefully pave the way for many such teaching sessions in the future.

## Appendices

Feedback form

Please assign a score of (1-5) with 1 being least likely and 5 being most likely. Include your comments as well for the questions.

Domain 1 - Planning of the Session

Item 1 - Were the learning outcomes achieved?

Item 2 - Was there adequate checking and assessment of prior knowledge?

Domain 2- Communication Skills

Item 3 - Did the facilitator break ice and open the session well?

Domain 3 - Quality of the Teaching Material

Item 4 - Were different modalities used to facilitate the session?

Item 5 - Were the slides legible and easy to follow?

Item 6 - Were the concept and facts broken down adequately and built up from previous knowledge?
